# Evolving from public health libraries as a place to focus on public health librarian expertise

**DOI:** 10.5195/jmla.2024.1804

**Published:** 2024-04-01

**Authors:** Kristine M. Alpi, Kayla M. Del Biondo, Melissa L. Rethlefsen

**Affiliations:** 1 krisalpi@gmail.com, Associate Dean of Libraries & Information Sciences, Icahn School of Medicine at Mount Sinai, New York, NY; 2 kayla.delbiondo@yale.edu, Simbonis Librarian for Public Health, Harvey Cushing/John Hay Whitney Medical Library at Yale University, New Haven, CT; 3 mlrethlefsen@gmail.com, Executive Director & Professor, Health Sciences Library & Informatics Center, University of New Mexico, Albuquerque, NM

**Keywords:** Public health, Academic libraries, Library spaces, Branch libraries, Health departments, Public health librarianship

## Abstract

**Objective:**

This article describes the evolution of academic public health library services from standalone academic public health libraries in 2004 to centralized services by 2021.

**Methods:**

Five public health libraries serving public health graduate programs (SPH) at public and private institutions were visited in 2006-07. Visits comprised tours, semi-structured interviews with librarians and local health department staff, and collecting of contemporary print documents. We compiled and compared visit notes across libraries. In 2022, we reviewed online materials announcing library closure or transition for timing and how services were to be subsequently provided.

**Results:**

Libraries and SPH were co-located and most librarians maintained public health expertise though they did not have faculty appointments in their SPHs. Specialized statistical and geographic information systems (GIS) software and data were provided in partnership, often with other system libraries. Only two libraries had strong connections to health departments–one with direct service agreements and another engaged in public health training.

**Conclusion:**

Academic public health libraries' relationships with SPHs and health departments did not ensure their existence as standalone entities. Following a national trend for branch libraries, public health information services were centralized into larger health or science libraries. The scope and specialization of librarian expertise continues to be valued with several institutions having librarians dedicated to public health.

## INTRODUCTION

In 1955, Flora Herman, then librarian of the Florida State Board of Health, reported that at the time in the United States, there were “probably eight states” without a public health library, four of which were planning for a library in the following two years [1]. Herman stated, “[m]arked variation is noted among libraries even within the same field. The purpose, clientele served, the physical setup, and, last but not least, the staff of each individual library contributes to this dissimilarity” [1]. Public health libraries (PHLs) include health department or agency libraries as well as academic public health libraries (APHLs) affiliated with schools or programs of public health (SPH).

At the 50th anniversary of Herman's article, in 2005, there were few standalone APHLs in the United States and the number of state health department libraries/resource centers had dwindled, with only 24 having a public-facing online presence [2]. Numerous academic health sciences libraries received funding from the National Library of Medicine to support and partner with public health agencies (e.g., state or county health departments that lacked their own libraries) [3–7].

The original project sought to understand how APHLs functioned in order to apply this knowledge to the improvement of libraries and information services for local and state departments of public health. The objectives were:

1. to quantitatively and qualitatively assess commonalities and differences among APHLs;
2. to explore the relationships between APHLs and the public health agencies that receive their students, as well as the interplay between these libraries and their counterparts in public health agencies and national organizations; and
3. to disseminate information on public health libraries to both library and public health audiences.

As the project was being conducted and written up, major changes were occurring within the participating APHLs as well as the larger library community [8–10]. Thus, it was important to track and understand how APHLs evolved. Therefore, secondary objectives were:

1. to contrast the perspective gained from visiting the individual APHLs to the current state of spaces and services in those institutions; and
2. to report how one of the APHLs transitioned to a service model not dependent on location.

## METHODS

In 2004, working in a municipal health department, the first author (KMA) began a Medical Library Association (MLA) Kronick Travelling Fellowship to better understand APHLs [11]. She proposed this to the bureau director as a group of site visits for informing local practice, a method that was common in agencies. The agency's Institutional Review Board (IRB) was not consulted for this presumed quality improvement (QI) project. In 2022, when the project transitioned from internal QI to external knowledge sharing, we contacted the library directors involved. They each checked this manuscript and agreed to the final version.

### Positionality Statement

All three authors practiced public health librarianship. KMA directed a local public health agency library from 2003 to 2005. MLR was a state public health department library staff member and librarian from 2001 to 2005. KMA and MLR belonged to the Public Health/Health Administration (PH/HA) Section of the MLA. KDB is a public health liaison librarian who received the Sewell Stipend [12] to attend the American Public Health Association (APHA) Annual Conference.

### Participating Libraries

The five standalone APHLs approached were the Epidemiology and Public Health Library (also called the Ira V. Hiscock Public Health Library) at Yale University (Yale), the Sheldon Margen Public Health Library at the University of California, Berkeley (Berkeley), the Abraham M. Lilienfeld Library at Johns Hopkins University (Hopkins), the School of Public Health Library at the University of Texas Health Science Center (UTHealth) at Houston (Houston), and the Public Health Informatics Services and Access (PHISA) at the University of Michigan (Michigan) [13]. Four were selected as they represented four distinct areas of the country and served longstanding schools of public health. The fifth library was added as it was geographically convenient.

APHLs existed in a larger environment, including the academic and academic library community with government documents or GIS services as well as the larger public health community. The visits to Berkeley and Michigan included structured interview meetings with local or state public health agencies to assess their view of their information services and training.

## DATA COLLECTION

### Phase 1: Kronick Visits

The project plan was to gather basic organizational information, statistics on budget, workforce composition, scope of operations, mission and vision, organizational structure, and organizational history, combined with site visits to provide impressions of organizational culture [14]. Additionally, KMA would review documents such as maps of the library, collection development policies, and other procedures that may be of assistance to other PHLs in developing their policies and procedures.

Prior to the visit, the library directors were asked to complete an open-ended questionnaire (Appendix A) about collections, facilities, services, staffing, training offered, relationships with state and local public health agencies, community-based organizations, and state and local libraries, as well as participation in national organizations. The questionnaire drew on MLA Benchmarking questions [15], the issues assessed by Herman's questionnaire [1] and KMA's experience in PHLs. The library directors were asked for permission to analyze and share their data in aggregate.

Between February and April 2006, KMA made one or twoday visits to each of the participating libraries. Itineraries were customized based on discussion with the PHL and the availability of internal and external partners. During those visits, KMA took notes on her observations, gathered printed materials such as brochures and flyers, and completed semi-structured interviews with the library director and other members of the library staff as available, particularly those serving the public health community. In addition to written notes, several of these interviews were also recorded; the audio files were saved but not transcribed.

The first author (KMA) analyzed data from handwritten notes, audio files, brochures, and flyers. She compiled data from the questionnaires and compared data across the other participating libraries looking for similarities and differences. The sections and language from the questions (Appendix A) provided the organizing structure for the initial analysis. She refined the names of categories containing the collocated clusters of responses. As the data compilation and analysis were underway, the status of these libraries began to evolve with shifts to other service models and closing or merging into other libraries.

### Phase 2: Academic Public Health Library Status in 2022

All five physical libraries no longer existed in 2022. We sought records of what happened to each library and its services using a combination of searching Google for the library names, searching their affiliated SPH and/or academic library system websites directly, and using Internet Archive to locate news items regarding each library's merger or transformation. We captured relevant documents about each library for contemporaneous news as well as historical narratives that touched on the libraries. We sought the years associated with each library's opening and closure, as well as the founding date of each affiliated SPH. Michigan's closure of the public health branch library and transition to a team-based support model for public health was included in a broader article about the transformation of their Taubman Medical Library into the Taubman Health Sciences Library [16].

As the public record for other AHPLs was sparse on the process and motivation for transitioning from the physical location to the current service model, a public health librarian from Yale (KDB) who investigated the presence of peer library services on SPH websites [17] joined the project to extend the story of one of the participating libraries. To provide richer insight, she worked with local colleagues to track down additional details and situate the earlier findings in the context of how public health information services were presented online in 2022 by their SPH partners producing a case report of how Yale shifted in library services from 2004 to the present day.

## RESULTS

### Findings from the In-Person Visits

Table 1 provides data about the participating libraries and their associated SPH. The substantial variation across libraries is shown by both the numeric data and in the examples in each category of the basic analysis.

**Table 1:** Participating Academic Public Health Library Data, 2005-2006. Data provided by participating libraries in either 2005 or 2006 depending on time of visit; some amounts estimated or provided from review of annual reports.

University (Status)	Public Health School/Program Start Year	Library name	Library Start Year	Medical or Academic Health Sciences Center Library on Campus	Staff FTE	Square Footage	2005–06 On-Site Volumes	Journal Subscriptions	Primary Faculty	Graduate Students
California, Berkeley (Public)	1943	Sheldon Margen Public Health Library	1947	No	7.1 (includes contract sites)	8,746	99,291	1,173	64	516
Johns Hopkins (Private)	1916	Lilienfeld Library	1967	Yes	2.0	Not available	31,500	200	485	1,949
Yale (Private)	1915	Ira V. Hiscock Library	1945	Yes	3.0	3,500	25,000 monographs; undetermined number of bound journals	350	58	266
Michigan (Public)	1941	Public Health Library & Informatics	1943	Yes	10.5	2,932	106,556	424	120	840
Texas at Houston (Public)	1967	School of Public Health Library	1970	No	4.5	9,535	56,795	320	146	1,073

### Library Identity, Reporting, Budget, and Staffing

Unique collections and staff expertise were reasons for establishing an APHL. This response from Johns Hopkins, quoted with permission, provides more insight:

The Interdepartmental Library was established to meet the specialized information needs of the students of the School of Hygiene and Public Health, needs which it was felt were not being met by other Hopkins Libraries. The new library's collection was interdisciplinary in nature, reflecting the varied backgrounds and interests of the School's students and faculty. Its collection was inherently less clinical in nature than the other libraries on the Medical Campus. For example, there has always been a strong focus on collecting works pertaining to the social and political aspects of health care [18].

While the APHL identity was important, they also benefited from identifying with the larger university library system. All of the libraries except Houston were considered libraries in the larger university system as they related to online resources and journal licensing. At Houston, the university contracted with the Texas Medical Center Library to provide library services and resources. Thus, all APHL constituents had access to a wide range of library resources and services in addition to those provided by the APHL. Three of the libraries operated on a campus with a larger academic health sciences library presence.

This relationship with other campus libraries evolved over time for some APHLs. At Johns Hopkins, it was not until 1996 that the Lilienfeld Library became a branch of the Welch Medical Library. In most cases, the APHL leader formally reported through the university library structure–to the Medical Library, an Assistant University Librarian, or some other title. Only one library reported within the SPH, to the Associate Dean of Research, and that library had informal reporting also to the system library director. At least one library reported having a dotted line to the Dean of SPH to make it easier to respond proactively. When asked about advisory groups or committees, one APHL shared that their library committee no longer existed because the school preferred not to have a formal group.

The budgets reported were typically for collections uniquely purchased by the APHL ($95K - $159K) and did not include the salaries of the APHL staff. In at least one case, the budget for the library came from the SPH even where the formal reporting was not to the SPH. In the libraries without additional contract staff, staff size ranged from two to five FTEs of which one to three were librarians; several libraries reported part-time graduate assistants, including one library also supervising the statistical software teaching assistants. In addition to the staff specific to the APHL, there were often librarians or informationists with at least a part-time focus on public health at the medical library. Additionally, many of the university libraries had librarians focused on GIS/mapping, data and statistics, and government documents who provided services and resources for those areas critical to public health practice.

Librarians had substantial expertise in public health, including public health or related degrees or APHA membership or participation in national, state, or regional public health activities. APHA annual meeting participation, often with support from the Sewell Fund [12], was important to developing public health knowledge and making connections. None of the librarians reported having SPH faculty appointments/status, despite of having substantial teaching roles in the public health curriculum.

### Library Presence, Specialized Content and Expertise, and Services

All APHLs had their own websites. At universities where there was not a larger medical library, the APHL website was linked from the SPH website and, in Houston's case, on the top toolbar across the entire organization's site, as it was the lone library for that organization. The website of the library that provided contracted services to health departments was reportedly linked from the Intranet of one health department under a category labeled Workplace Tools.

All libraries used central technical services such as the shared online public access catalog. Though APHL staff participated in university library structures for technical services, at least two wished for more ways to engage with those colleagues. For interlibrary loan (ILL), all but one used central services from the university or medical library. The one with its own presence in DOCLINE, OCLC, and local resource-sharing consortia reported unsuccessfully joining Libraries Very Interested in Sharing (LVIS), the first global OCLC no charge Resource Sharing Group. They learned quickly that LVIS libraries did not have the materials SPH needed, and they were inundated with requests.

### SPH Location and Library-SPH Collaborations

**Figure 1:**
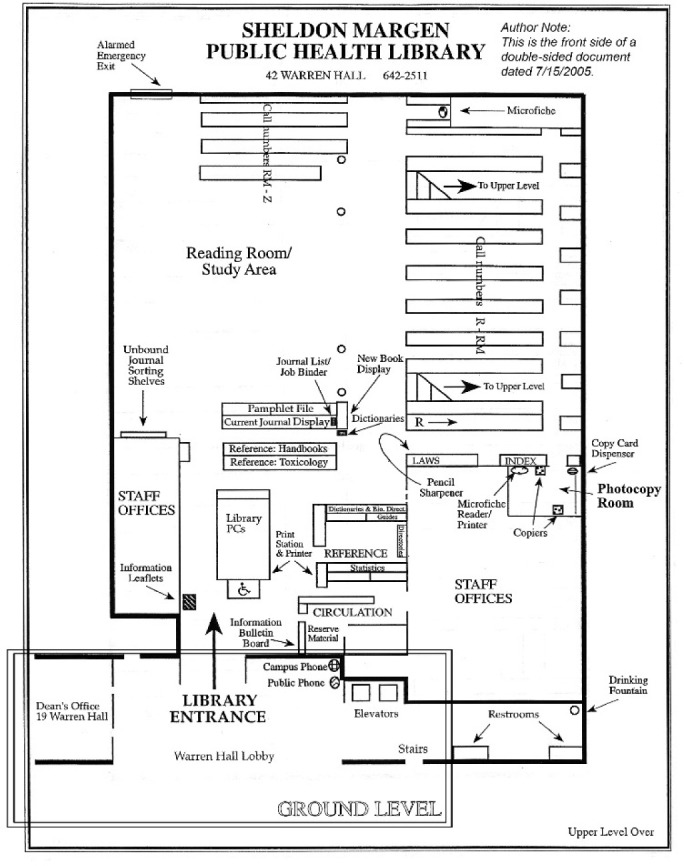
First page of the Berkeley Public Health Library Floor Plan. Note: the outline around the entrance location was added for the purpose of this article.

All APHLs were located within a building that included other SPH academic services, often having prime visibility on the first floor of the main SPH building. Figure 1 shows the first-floor entrance for the Berkeley Library as an example. Two libraries described touchdown stations in other buildings on the same campus where public health researchers were located. In one case, this meant an additional satellite library directly in SPH and touchdown stations by the SPH digital lab. In another, there were two locations in the health department buildings that housed contracted partners. The locations were not where the libraries had always been or where they were planned to be; plans to move the library to accommodate adding an undergraduate public health major to the school was mentioned by one library.

**Figure 2:**
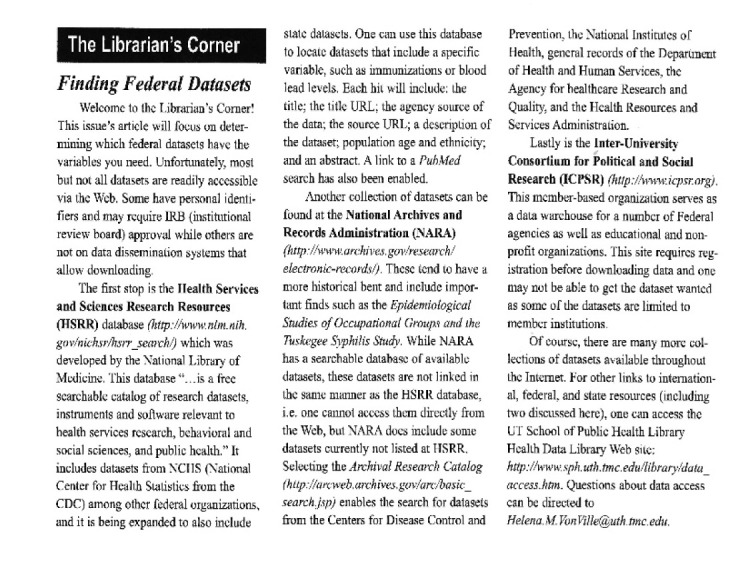
The Librarian's Corner column on “Finding Federal Data Sets” from the Spring 2006 issue of news@tphtc, published by the Texas Public Health Training Center.

Coordination between the APHL and the SPH was common in instruction and the provision of specialized software and data services. In most, librarians were invited by the SPH instructors to teach literature searching and information management skills in the curriculum as well as participate in orientations with a median of 25 sessions annually. In another APHL, new student searching courses were taught by the larger medical library and so rather than teach courses, the APHL provided consultation appointments. One of the libraries also provided faculty development on instruction and digital tools. Two of the libraries co-managed a computer lab in the library with specialized statistical software and in other cases, the statistical lab was nearby in the school building. One library reported that the SPH was working on developing a virtual statistics and software lab. Figure 2 shows an article about data and statistical sources written as part of the regular librarian column in the Texas Public Health Training Center newsletter. The location of GIS and data services, library services, and instruction depended on the university library structure—map libraries, social sciences libraries, and main libraries were all points of referral.

Faculty publications were another area of library-SPH collaboration, and there were a variety of approaches for engaging with the Dean's office in this area. Where there was a mandatory annual faculty report to the Dean, the university maintained the faculty publications list and the library bought the faculty-published books. Another library reported buying faculty books and doing displays of faculty publications. Others set up searches for the Dean's office and covered new publications in the Faculty “News” section of the SPH site.

### Local and State Health Department Relationships

The largest difference among APHLs was the relationship with the local or state department of health. While it is not surprising that two private university APHLs had no official relationships beyond the occasional letter of support for grants or outreach projects, the three public university APHLs each had very different relationships. All the public university libraries were open to external researchers, so health department staff could receive assistance with research questions and use library computers onsite with a guest login. It was not possible to describe these models without identifying the libraries, so we requested permission from the participating libraries for the information shared in this section.

**Figure 3:**
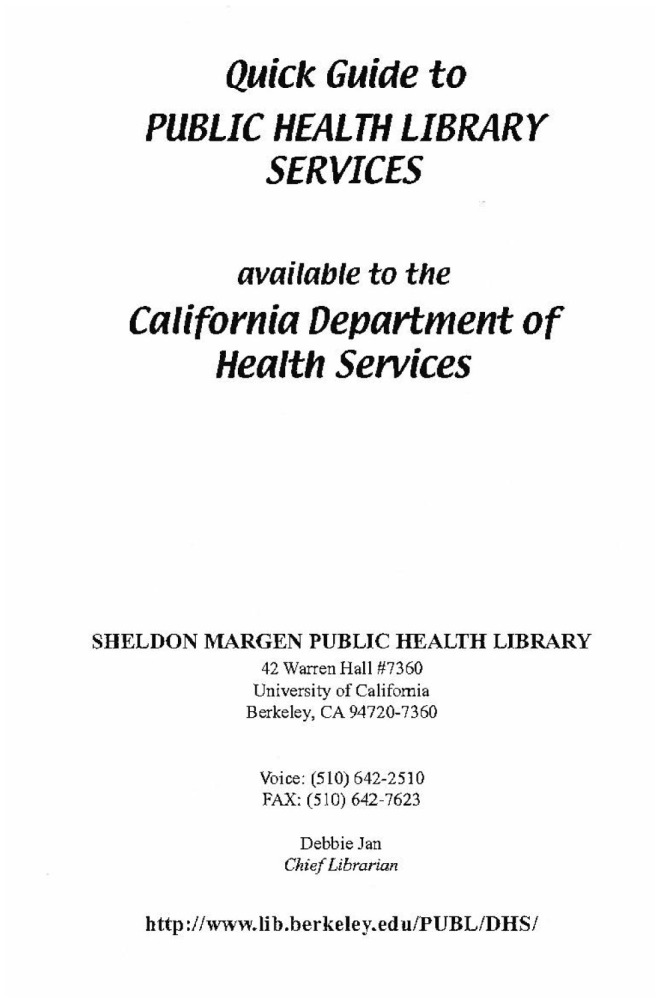
Front page of “Quick Guide to Public Health Library Services available to the California Department of Health Services” dated November 29, 2005.

In the case of Berkeley, the California Department of Health Services (DHS) collaborated in developing the SPH and gave its physical collection to form the Public Health Library, which then maintained journal subscriptions for DHS. At the time of the visit in 2005, there had been a contract for direct library services since 1955. Figure 3 shows the title page of the brochure about services available to DHS in 2005. Its eight section headings covered Training, On-Site Use, Mail Delivery, Library Cards, Reference Services and Literature Searching, Current Awareness Services, Document Delivery, and Electronic Journals.

The relationship between Michigan's PHISA and the Department of Public Health included librarian-led training. For example, Michigan librarians taught a full-day course on “Retrieving Online Information” in Traverse City for the Great Lakes Intertribal Council which was co-sponsored by the Michigan Center for Public Health Preparedness and the Michigan Public Health Training Center.

The youngest of the participating libraries, Houston, did not have a relationship with the local or state health departments, primarily due to the prior establishment of other library support for those entities. The Texas State Department of Health has its own library, and the Houston Academy of Medicine-Texas Medical Center (HAM-TMC) Library provided services to the city and county health departments through the Houston Department of Health and Human Services. However, the Houston librarian did share information about relevant resources with the Texas Public Health Training Center as shown in Figure 2.

### Last Words—What APHLs Wished for in 2005

The interviews closed by asking, “What is one thing you wish you had funding for?” While unsurprisingly all of them wished for more librarians and funds, these small libraries were also visionary. The most ambitious wish list had both internal and external components. Internally, these included improving the public workstation space, having a training room within the library, supporting students who do not buy their textbooks, providing more databases, a process for digitizing dissertations, building more interaction/marketing into the course management system, working with faculty on curriculum-integrated instruction and course readings, and being better aligned to research efforts. They also wished for better networking among librarians and vendors that support short-term pay-per-use. Externally, they wanted to do more community outreach, have another librarian for outreach to the regional campuses, and have travel money in their budget.

### Summary of Similarities and Differences

All libraries were focused primarily on public health as a discipline and offered physical proximity to the SPH, with librarian expertise and professional engagement in public health organizations. All but one of the libraries operated in an academic environment where there was a broader health sciences or medical library on campus providing some collections and services. The provision of specialized statistical and GIS software and source data varied depending on whether the computer lab with the statistical and mapping software was in the library. Differences were primarily with the SPH and local or state health department relationships. Only one had a contract for direct service and staffing. Unofficial areas of agency-librarian cooperation included emergency preparedness and public health informatics training, all of which were stronger in public institutions.

### Findings from the Libraries Status in 2022

**Figure 4:**
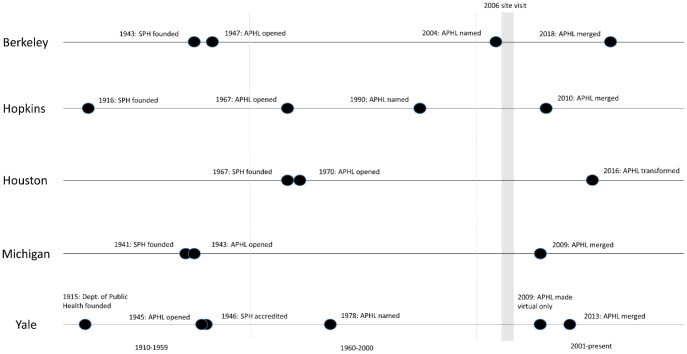
Timeline of sampled APHLs. Four libraries merged between 2009 and 2013. Houston transformed into a learning resource center in 2016 and had no professional librarian as of 2017. Note: The thin vertical lines separate the three time periods in the figure (1910-1959, 1960-2000, and 2001-present. The thick vertical line represents the time frame of the site visits.

Figure 4 shows a timeline of the libraries' launch, naming, and cessation based on publicly available announcements. Three libraries were ultimately named for famed public health faculty members at their institutions, all substantially after their foundation. The library with the longest operating history was Berkeley, which launched in 1947 and was renamed for one of UC Berkeley's emeritus faculty, Sheldon Margen, in 2004, shortly before his death. Berkeley merged into the Marian Koshland Bioscience, Natural Resources & Public Health Library in 2018. The earliest named library, the Ira V. Hiscock Library at Yale, was named for a former department chair in 1978 and was extant until 2008. Johns Hopkins' Lilienfeld Library was named in honor of long-time faculty member Abraham M. Lilienfeld in 1990. It closed in 2010 with services transferring to the Welch Medical Library.

Michigan was the earliest APHL created in 1943 with the creation of the SPH and going through evolutions to incorporate informatics before being absorbed into the Taubman Health Sciences Library in 2009 [13, 16]. The youngest library was the UT Health School of Public Health Library (Houston), whose first library director was hired in February 1970 and subsequently established the library. Beginning in 2016, Houston's library transformed into the SPH Library & Graduate Communication Center, retaining the word “library” in the new name, but no longer employing professional librarians. In approximately 2019, Houston's SPH contracted with the Texas Medical Center Library to provide librarian services half-time.

### Case Report: Library Support for Yale School of Public Health, ‘Then & Now’

In a 2014 study that explored the information needs of public health students, Le concluded that librarians serving public health students must perform targeted marketing to constituents to leverage relevant library services and build relationships [19]. There is also literature that links library visibility on professional schools or departments' websites to the library's involvement in research projects and library use as a whole [20–23]. This is the case for Yale, where library services for public health have moved from physical embeddedness to embeddedness achieved through mindful marketing, relationship building, and digital presence.

The APHL at Yale closed in 2008 during the financial crash and transitioned to an online-only library [24]. The online-only library closed in 2013, and, from then until 2016, there was no dedicated public health librarian. In 2016, a librarian was hired to support the school. Since being hired, she has co-authored evidence synthesis and meta-research papers, been involved in an SPH committee, and was appointed as a lecturer in an SPH department in 2019.

In 2021, in response to increased student enrollment in SPH programs, KDB was hired as the Simbonis Librarian for Public Health, a three-year term position for a librarian primarily supporting Yale's SPH. With more library staff to support Yale's SPH, the public health librarians became more visible to leaders and were able to think more strategically about outreach to engage with constituents in new ways [25]. They have been invited to lecture during non-curricular happenings including student case competitions and research fellowship informational sessions for students, and they have been asked to be advisors for grant proposals written by SPH Department Chairs. Additionally, they are compiling findings from a primary research study exploring SPH faculty members' understanding of open access publishing and received great support from SPH leadership while conducting this study.

Some aspects of 2004 Yale library services for public health are not much different from present day. There is still no formal partnership between the medical library and the New Haven Department of Health or the Connecticut Department of Health, though the medical library is open to the public. The public health librarians are active in professional associations and conferences, including APHA. They do not formally track SPH faculty publications but have helped SPH leaders create searches to identify faculty publications for annual reports. The organizational structure has remained such that public health librarians are a part of the larger Yale University Library promotion/ranking system and report to the Cushing/Whitney Medical Library Director. While KDB's position is currently funded through an endowment, the cost of the other public health librarian is paid for by Yale School of Medicine, of which SPH is a department, at the time of this writing.

Unsurprisingly, the Yale web presence has also evolved. In 2004, the APHL had its own website, which was available directly from the SPH website and the medical library website. While this website no longer exists, the public health librarians launched a ‘support for Public Health' webpage on the medical library website in 2022 and phased out existing public health LibGuides, which were used from 2016-2021. They asked the SPH Marketing and public relations team to link to this webpage from the SPH website, to which they agreed.

### Liaison librarian visibility on SPH websites

Del Biondo assessed public health liaison librarian visibility on six schools of public health websites and found that only two of the six schools mentioned the medical library that serves the SPHs on its websites [17]. This method was used to evaluate the visibility of library services for SPHs on the websites of the SPHs included in the original Kronick study and the number of clicks to reach the medical library's website was also recorded [23]. This website analysis was conducted in June 2023 and represents the SPH website layouts at that specific point in time. Table 2 summarizes these observations.

**Table 2:** Portrayal of library services on six peer institution SPH websites as of June 2023

Name of SPH	Is the Library that serves the SPH indicated on the SPH website? If ‘yes,' through which menus/navigation?		Are public health librarians mentioned on the SPH website?	Number of clicks it takes to get to the Library website from the SPH website	Name of the library that serves the SPH	Number of public health librarians
UC Berkeley School of Public Health	Yes	Under menu → ‘Student Life’ → ‘Marian Koshland […] Library’	No	2	Marian Koshland Bioscience, Natural Resources & Public Health Library	1
Johns Hopkins Bloomberg School of Public Health	No	The website doesn't indicate which library serves the SPH; although, all campus libraries are listed in the footer menu of the homepage under ‘Offices and Services’ → ‘Libraries’	No	3	William H. Welch Medical Library	2
University of Michigan School of Public Health	Yes	Under ‘community’ → ‘student experience’ → ‘campus resources and services’ → ‘University Libraries’	No	3	Taubman Health Sciences Library	1
UT Health Houston School of Public Health	Yes	In footer menu of homepage under ‘Quick Links’ → ‘Library’	No	2	Texas Medical Center Library	1
Yale School of Public Health	Yes	Under menu → ‘Admissions and Financial Aid’ → ‘Student Life & Academic Support’ → ‘Libraries at Yale’	Yes	4–5 The link to the library is listed on ‘Student Life & Academic Support' page and ‘Libraries at Yale' page	Harvey Cushing/John Hay Whitney Medical Library	2

Arrows indicate navigation to named webpages or sections. All clicks were counted, even if it simply expanded a menu.

Although the physical APHLs have closed since the Kronick visits, four of the five SPHs' websites highlight library services or collections for public health and/or note the medical library which serves public health. This may be to market to prospective students in addition to serving as a resource or reminder to current students, faculty, and staff. The one Hopkins SPH does not describe library services, however, the school links out to the university library system from its website. In the case of Yale, the public health liaison librarians are noted on the SPH website's description of the Library. For the other schools, the library website is hyperlinked so that a web user could find additional information upon visiting the website and exploring (e.g. searching for staff or selecting a LibGuide by subject area). According to the Welch Medical Library web sites (Hopkins), one informationist serves Public Health Administration and one serves Public Health Graduate Students. In the case of Michigan's Taubman Health Sciences Library website, there is a Global Health Coordinator for Library Research not accounted for in Table 2.

## DISCUSSION

### Limitations

We are not able to share the detailed interview notes and findings or attribute most of them to individual libraries due to the original agreed-upon internal QI-only nature of the interviews. If these investigations were being pursued today, we would have engaged with the IRB and developed a prospective informed consent process for all those interviewed. The health department perspective is also limited as we did not meet with the health departments in Houston, Baltimore, and New Haven, as the librarians indicated that there was not a service model or collaboration.

### Special Services Require Expertise

The primary finding at the time of the visits was that APHLs were in a great state of flux. Those located at universities with medical centers were being further integrated into the overall medical library with a primarily virtual collection and less emphasis on physical collections and spaces as the typical branch library model. They were moving from being supported by a small PHL staff to being served by the reference and outreach efforts of the larger academic library, often by integrating the public health librarian into the larger staff. These findings are institution-specific and neither these reports nor the website analysis are intended to suggest that they fully represent the larger picture of PH library services and expertise in the United States.

One united health sciences library as opposed to a standalone public health library has its strengths, judging by Yale's experience. When liaison librarians are close to the larger library's stacks, technologies, reservable spaces, and historical collections, this fostered a greater understanding of the library's diverse collections, so that liaisons can confidently organize programming and tours for their constituents in one centralized place. This also allows librarian colleagues to more readily collaborate, ask each other for assistance, and answer general reference questions or directional questions from other schools and disciplines.

### Public Health Knowledge Resources Access in a World without APHLs

The hope was that these visits would lead to increased communication and collaboration among academic and governmental public health libraries, but as budget challenges focused academic libraries on their core constituencies of faculty, staff, and students, the responsibility to be a state or local resource was less of a priority. Collection access was one of the only avenues for health department staff to use the libraries. As APHLs were dismantling their print collections and dealing with back issues, storage, and digitization, it is unclear what happened to these collections, except for Yale, where 2089 volumes from 58 titles from the APHL were absorbed into the Medical Library collection. This absorption of subject-specific branch libraries is not unique to public health libraries. Our findings parallel a larger trend in the closure or consolidation of branch libraries in academia. In some cases, the spaces remain as study centers with the staffing and collections returned to the main library. In other cases, the physical footprint has been absorbed entirely [8].

Public health journal coverage online has improved, and *American Journal of Public Health (AJPH)* and *Public Health Reports* have digitized backfiles in PubMed Central. However, as we learned from public health bibliometric studies of public health nursing [26], environmental health [27], infectious disease [28], and *AJPH* [29], the older literature is still cited. Many public health departments now participate as members of the Public Health Digital Library [30], which provides a core collection of online resources and partners libraries with an academic health sciences library for ILL services. Very few state or local public health libraries in the United States, such as the Texas Department of Health Library, remain.

During the COVID-19 pandemic, the world witnessed huge demands for public health professionals and evidence-based public health information. Public health professionals must understand how to access and use information, and librarians are critical to their professional development. The closure of physical libraries does not reflect the demand for public health librarian expertise, which remains strong, especially as academic public health programs continue to expand. Continuing to invest in public health librarian expertise is essential for SPH and the libraries that partner with them.

## CONCLUSIONS

The nuances in comparing five APHLs, much as Herman had commented in 1955, were complicated enough to shift from quantitative reporting on the focused questions in the interview guide to qualitative description beneath broad categories. The lessons learned from standalone PHLs still inform the practice of this generation of librarians working in these spaces who have successfully transitioned to the current model of centralized library services to public health. However, these librarians are aging out of the profession making it timely to reflect on these experiences and document the history of these libraries in supporting public health learners and workers.

Regardless of physical location, librarian outreach can be successful through strategic communication and relationship-building that deepens over time. We are optimistic about the value that public health librarians and informationists demonstrate. The large number of MLA members engaged in the Public Health/Health Administration Caucus (261 as of December 14, 2023 [31]) suggests that this expertise continues to be developed and deployed to educate those who will ultimately improve the health of the public.

As was intended for the original project, this article is one attempt to communicate awareness of public health librarian practice and services in a venue that is accessible to public health educators and decision makers who fund public health workforce development, such as agency and university administrators responsible for meeting the learning needs of those in public health educational programs. We hope our work sparks conversations and collaborations among public health leaders and librarians who support public health about what combination of library support models and services ensure successful learning experiences that prepare public health practitioners for their wide-ranging and critical roles in our society.

## FUNDING

The site visits described in this project were funded by the Medical Library Association Kronick Traveling Fellowship in 2005 (now known as the MLA David A. Kronick and Charles W. Sargent Visiting Fellowship).

## DATA AVAILABILITY STATEMENT

Additional data from this quality improvement project is not publicly available because participants were not asked to provide consent to have their information shared for research purposes.

## AUTHOR CONTRIBUTIONS

Kristine M. Alpi: conceptualization; formal analysis; funding acquisition; investigation; methodology; project administration; writing – original draft; writing – review & editing. Kayla M. Del Biondo: investigation; writing – original draft; writing – review & editing. Melissa L. Rethlefsen: conceptualization; investigation; project administration; visualization; writing – original draft; writing – review & editing.

## SUPPLEMENTAL FILES



**Appendix A**
