# Supplementary material for: Evolving from public health libraries as a place to focus on public health librarian expertise
Source: J Med Libr Assoc. 2024 May 22;112(2):95–106. doi: 10.5195/jmla.2024.1804 (PMC11305463; doi:10.5195/jmla.2024.1804)
Supplement: Supplementary file 1 — Appendix A [file jmla-112-2-95-s01.docx]

Appendix A: **Kronick Travelling Fellowship Inquiry**

**Background**

What is the purpose or mission of your library?

How long has your public health library been in existence?

What is your library’s relationship with the local or state department of health?

What is your organization’s relationship with the local or state department of health?

**Clientele**

What clientele is served by your library? [see specific questions below]

How many MPH students (part-time and full-time)?

How many PhD students (part-time and full-time)?

How many undergraduate students?

Any other types of students?

Do you serve local health department staff?

How many FTE at your local HD?

How far is your library from the local central HD office?

Does the HD have its own library? If not, is it served by the state library?

Do you serve state health department staff?

How many FTE at your state HD?

How far is your library from the state central HD office?

Does the HD have its own library? If not, is it served by the state library?

Are any of the above services free? If not, do you have a contract with the health department? Are the services available as pay per use? Please elaborate on whatever situation is in place.

Do you serve alumni?

Do health department staff serve as visiting/guest lecturers? And if so, do they have extended privileges?

What is your relationship (current or past) with the various information centers of the Centers for Disease Control & Prevention?

How many items did you circulate last year?

Is your library open to the community?

Do you participate in interlibrary loan via DOCLINE? OCLC? Are you a FreeShare or LVIS library? Do you participate in local resource sharing consortia?

How many items did your library lend last year to other libraries?

**Organization**

To whom does your library report administratively?

Where is your library located physically? Standalone, in the school of Public Health, in a larger library?

Does your library have satellite offices or program libraries or touch down stations?

Does your library have its own web site?

Where is your library’s web site linked from your larger organization?

Do you keep your entire collection in-house? What is the cut-off year for journals or do you keep all your back runs? Do you have space in a remote shelving unit?

Do you have a history/rare book/archives collection?

How many seats in the Library?

How many computers available in the Library?

Does your library have a computer lab? Library-managed, shared with university?

Do curriculum-based courses such as GIS and Biostatistics get taught in the Library computer lab?

**Collections**

Is your online public access catalog standalone or shared? Is it available to all? Do you participate in OCLC WorldCat?

How many current journal subscriptions do you receive?

What % or number of those include online access?

How many volumes are in your collection?

What is your annual collections budget?

How many books did you add to the collection last year (either calendar or fiscal)?

How many A/V or software items did you add to the collection last year (either calendar or fiscal)?

Are you an international/federal/state or local depository library? If not, do you collect international/federal/state or local public health documents? Mark all that apply. Is there a government documents depository library near your library? What is your relationship to that library?

How many databases do you make available to your users?

How many items did you borrow through interlibrary loan last year [include items requested from other campuses]?

What are your loan policies to other public health libraries?

**Services and Staffing**

How many staff do you have and in which categories or departments?

How many of your staff members belong to library organizations? Public health organizations? Which ones?

How many reference questions do you respond to each year?

How many mediated searches do you conduct each year?

How many instruction sessions were offered by your library this year?

What is the annual usage of your self-service photocopiers?

Do you track and promote faculty publications?

Do you offer any update services or SDIs or journal TOC services?

Did you participate in teaching in the PH school curriculum this past year?

What is the overall budget for your library?

What is one thing you wish you had funding for that could improve the services offered by your library?
